# The BEL1-like family of transcription factors in potato

**DOI:** 10.1093/jxb/ert432

**Published:** 2014-01-27

**Authors:** Pooja Sharma, Tian Lin, Carolina Grandellis, Mei Yu, David J. Hannapel

**Affiliations:** ^1^Plant Biology Major, Iowa State University, Ames, IA 50011, USA; ^2^Instituto de Investigaciones en Ingeniería Genética y Biología Molecular Dr Hector N. Torres, Consejo Nacional de Investigaciones Científicas y Técnicas, Buenos Aires, Argentina; ^3^Department of Plant Pathology and Microbiology, Iowa State University, Ames, IA 50011, USA

**Keywords:** BELL1, KNOTTED1, mobile RNA, *Solanum tuberosum*, TALE, tuberization.

## Abstract

BEL1-type proteins are ubiquitous plant transcription factors in the three-amino-acid-loop-extension superfamily. They interact with KNOTTED1-like proteins, and function as heterodimers in both floral and vegetative development. Using the yeast two-hybrid system with POTATO HOMEOBOX1 (POTH1) as the bait, seven BEL1-type proteins were originally identified. One of these genes, designated *StBEL5*, has transcripts that move long distances in the plant and enhance tuberization and root growth. Using the potato genome database, 13 active BEL1-like genes were identified that contain the conserved homeobox domain and the BELL domain, both of which are essential for the function of BEL1-type proteins. Phylogenetic analysis of the StBEL family demonstrated a degree of orthology with the 13 BEL1-like genes of *Arabidopsis*. A profile of the gene structure of the family revealed conservation of the length and splicing patterns of internal exons that encode key functional domains. Yeast two-hybrid experiments with KNOTTED1-like proteins and the new StBELs confirmed the interactive network between these two families. Analyses of RNA abundance patterns clearly showed that three *StBEL* genes, *BEL5*, *-11*, and *-29*, make up approximately two-thirds of the total transcript values for the entire family. Among the 10 organs evaluated here, these three genes exhibited the 12 greatest transcript abundance values. Using a phloem-transport induction system and gel-shift assays, transcriptional cross-regulation within the StBEL family was confirmed. Making use of the potato genome and current experimental data, a comprehensive profile of the StBEL family is presented in this study.

## Introduction

The BEL1-like family (BELL) of transcription factors is ubiquitous among plant species and they interact with KNOTTED1-like proteins to regulate a range of developmental processes (Müller *et al.*, 2001; Chen *et al.*, 2003; Smith and Hake 2003; Kanrar *et al.*, 2008; Lal *et al.*, 2011; Li *et al.*, 2012). These BEL1-like homeodomain (BLH) proteins have significant roles in meristem and floral development, and their functions are often overlapping and redundant. ARABIDOPSIS THALIANA HOMEOBOX 1 (ATH1), PENNYWISE (PNY), and POUNDFOOLISH (PNF) are BLH proteins of *Arabidopsis* that are critical for the initiation, maintenance, and development of the shoot apical meristem (Rutjens *et al.*, 2009; Ung *et al.*, 2011) and inflorescence architecture (Smith and Hake, 2003; Ragni *et al.*, 2008; Khan *et al.*, 2012). SAW1 (BLH2) and SAW2 (BLH4) are negative regulators of BREVIPEDICELLUS (BP), an important class I KNOX protein and positive regulator for growth. In the *saw1 saw2* double mutant, BP can be expressed on the margin of the leaf, and the leaf will develop with a serrated and revolute shape (Kumar *et al.*, 2007). Misexpression of BLH1 in the embryo sac will switch one of the synergid cells into an egg cell (Pagnussat *et al.*, 2007), and loss of function of the *AtBEL1* gene blocks the development of integuments (Brambilla *et al.*, 2007). In *Arabidopsis*, there are 13 BEL1-like family members, all of which can form heterodimers with KNOX proteins (Kumar *et al.*, 2007).

BEL1- and KNOTTED1-type proteins interact in a tandem complex to regulate transcription of target genes (Bellaoui *et al.*, 2001; Chen *et al.*, 2004; Hake *et al.*, 2004; Lin *et al.*, 2013). BEL1 also interacts with MADS-box transcription factors and SPOROCYTELESS to support ovule development in *Arabidopsis* (Brambilla *et al.*, 2007; Bencivenga *et al.*, 2012). POTH1 (*pot*ato *h*omeobox *1*) is a member of the class I KNOTTED-like homeobox proteins of potato. Using POTH1 as bait in the yeast two-hybrid system, seven BEL1-like proteins, designated StBEL5, -11, -13, -14, -22, -29, and -30, were isolated from stolon and leaf libraries of potato (Chen *et al.*, 2003). The heterodimer of StBEL5 and POTH1 exhibits a strong binding affinity to the promoter of *GA20ox1* and negatively regulates its expression (Chen *et al.*, 2004). DNase footprinting experiments identified the binding site of the POTH1–StBEL5 dimer in the *GA20ox1* promoter as a TTGAC double tandem motif. The TTGAC motif can be recognized by either POTH1 or StBEL5, but only when both TTGAC motifs are intact can the POTH1–StBEL5 heterodimer function (Chen *et al.*, 2004).

Several studies have demonstrated the role of StBEL5 and POTH1 in tuber development (Chen *et al.*, 2003; Rosin *et al.*, 2003; Banerjee *et al.*, 2009). Overexpression of each of these genes in transgenic potato lines produced plants that exhibited enhanced tuber yields. Heterografting experiments showed that the mRNA of *StBEL5* is mobile in both a downward and upward direction (Hannapel, 2013). Movement from leaves to stolon tips was enhanced under short-day (SD) conditions and mediated by the untranslated regions (Banerjee *et al.*, 2006, 2009). The mobility of *StBEL5* mRNA was dramatically reduced without the untranslated regions (UTRs), whereas a non-mobile mRNA exhibited increased mobility upon fusion with the *StBEL5* UTRs (Banerjee *et al.*, 2009). Besides enhancing movement of the mRNA, the UTRs also suppressed translation of a β-glucuronidase (GUS) marker in a transient expression system (Banerjee *et al.*, 2009). Recent mobility studies have also demonstrated movement of *StBEL5* into roots that impacts growth (Lin *et al.*, 2013).

From the recently published potato genome (Xu *et al.*, 2011), 14 BEL1-like loci have been identified including the seven original StBEL proteins isolated from the yeast two-hybrid screen (Chen *et al.*, 2003). Except for StBEL5, however, very little information is available on the other StBEL1-like family members. Transcripts of all seven of the original BEL1-like proteins were detected in RNA from phloem-enriched exudate or laser-captured microdissected phloem cells (Yu *et al.*, 2007; Campbell *et al.*, 2008). Making use of the reference potato genome (Xu *et al.*, 2011) and current experimental data, an extended analysis of the BEL1-like family of potato is presented in this study. Because of their functional relationship with KN1-like proteins and the potential for long-distance trafficking of their mRNAs, the *BEL1* genes of potato represent a valuable model for assessing the dynamic role these transcription factors play in plant development.

## Materials and methods

### Phylogenetic and gene structure analysis

A phylogenetic tree was constructed using the neighbour-joining method (Saitou and Nei, 1987) available in the MEGA 4.0.2 software package (Tamura *et al.*, 2007). Full-length proteins of the StBEL family were aligned using the ClustalW algorithm (Thompson *et al.*, 1994) included in the BioEdit software package (Hall, 1999). Gene expression data of StBELs for both the RH and DM genotypes was downloaded from Potato Genome Sequencing Consortium website (http://potatogenome.net). The *StBEL* genes were drawn to scale and assigned to potato chromosomes based on their positions shown in the PTGS (Release: Annotation v3.4, Assembly v3, Pseudomolecules v2.1.11).

### Real-time quantitative reverse transcription-PCR (qRT-PCR) for *StBEL* expression analysis

*Solanum tuberosum* ssp*. andigena* plants were soil grown for 4 weeks under long-day (LD) conditions and then transferred to either SD or maintained under LD conditions for a further 10 d. Leaf and stolon tip samples were harvested, frozen in liquid nitrogen and stored at –80 °C. RNA preparation and qRT-PCR were performed as described previously (Lin *et al.*, 2013). The relative gene quantification (comparative threshold cycle) method (Livak and Schmittgen, 2001) was used to calculate the expression levels of the *StBEL* RNAs. *StACT8* (accession number GQ339765) was used as an internal control. Products ranged from 98 to 160bp and were mostly designed spanning the introns in order to detect any genomic DNA contamination (Supplementary Table S1 available at *JXB* online). The specificity of primers was determined by melting curve analyses and agarose gel (3%) electrophoresis performed following the qRT-PCR experiments. A standard curve was generated based on six-point (10-fold) serial dilutions of cDNA to calculate the gene-specific PCR efficiency. PCR efficiencies of primers ranged from 97 to 110 %.

### Yeast two-hybrid system

The Matchmaker two-hybrid system (Clontech) was used for the yeast (*Saccharomyces cerevisiae*) two-hybrid screen with yeast strain pJ69-2A. The *StBEL* constructs were amplified by PCR and cloned into the vector pACT-AD (Supplementary Table S1 available at *JXB* online), in frame with the GAL4 activation domain. The tobacco *Knox* cDNA constructs were amplified by PCR and cloned into pBridge (Clontech) in frame with the GAL4-binding domain. Sequencing of selected cDNAs and constructs was performed at the Iowa State University DNA Facility, Ames, IA, USA. Positive interactions were confirmed by co-transforming into pJ69-2A with each purified pAD and pBridge plasmid and plating on –Leu/–Trp (transformation control) and –Leu/–Trp/–His/–Ade (selection) nutrient medium. Knox/StBEL interactions were quantified for *lac*Z induction using a β-galactosidase assay (Pierce Chemical). The Knox cDNA clones from tobacco (*NTH1*, *-15*, *-20*, and *-22*) were graciously provided by M. Matsuoka (Nishimura *et al.*, 2000).

### RT-PCR for *StBEL* RNAs in *GAS:BEL5* plants

Production and characterization of the *GAS:BEL5* transgenic line and verification of *StBEL5* RNA movement has been described previously by Banerjee *et al.* (2006, 2009). Transgenic or wild-type (WT) *S. tuberosum* ssp. *andigena* plants were grown under LD conditions for 3 weeks and then transferred to SD conditions for 10 d. RNA was extracted from leaves and roots and one-step RT-PCR was performed using 200–250ng of total RNA, a non-plant-sequence primer fused to the transgenic RNA, and a gene-specific primer for the *StBEL5* transcripts and a pair of gene-specific primers for *StBEL6*, *-34*, *-22*, and *-14* (Supplementary Table S1 available at *JXB* online). All PCRs were standardized and optimized to yield a product in the linear range. Homogenous PCR products were quantified using ImageJ software (Abramoff *et al.*, 2004) and normalized using 18S rRNA values.

### Gel-shift assays

Oligonucleotides with 3′ biotin labelling were synthesized at the DNA Facility, Iowa State University, Ames, IA, USA. dsDNA was prepared by hybridization of complementary synthetic oligonucleotides (Supplementary Table S1 available at *JXB* online). Gel-shift assays were performed using a LightShift Chemiluminescent EMSA kit from Thermo Scientific according to the manufacturer’s protocol with the following modifications. Twenty microliters of binding reactions were set up on ice containing 20mM HEPES (pH 7.5), 10% glycerol (v/v), 0.5% Triton X-100 (v/v), 0.5mM EDTA (pH 8.0), 50mM KCl, 2mM MgCl_2_, 20ng μl^–1^ of BSA, 1mM dithiothreitol, and 50ng μl^–1^ of poly(dI-dC) as a non-specific competitor. Ten femtomoles of labelled DNA was used for all assays. Two hundred nanograms of StBEL5–GST, 100ng of POTH1–GST or 200ng of glutathione *S*-transferase (GST) proteins were used (see Fig. 9). The binding mix was incubated on ice for 60min before electrophoresis. For the competition assays, unlabelled dsDNA fragments (100×, 200×, and 500×) were incubated with the recombinant protein on ice for 30min before addition of the labelled probe. Both the unlabelled and labelled DNA fragments used here were the same sequence.

## Results

### Phylogeny of the StBEL family

**Fig. 1.**
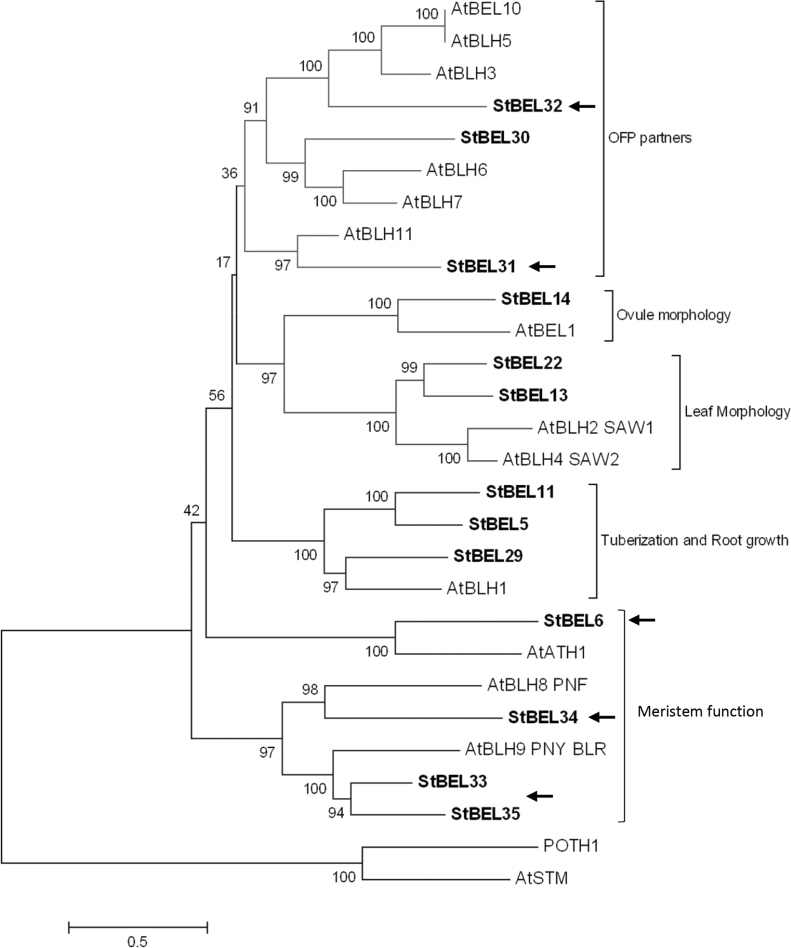
Phylogenetic relationship of the BEL1-like proteins of *Arabidopsis* and potato. The amino acid sequences of the 13 known potato BEL1-like proteins were analysed and compared with BEL1 proteins of *Arabidopsis*. These data were organized into a phylogenetic tree with the MEGA4.0.2 package and the neighbour-joining program. The numbers listed at the branching points are boot-strapping values that indicate the level of significance (percentage) for the separation of two branches. The length of the branch line indicates the extent of difference according to the scale at the lower left-hand side. StBELs are represented in bold letters. Putative functions are listed for each group. Arrows designate the six new StBEL proteins.

Originally, seven BEL1-like proteins were identified using the yeast two-hybrid system (Chen *et al.*, 2003). Additional sequences for BEL1-like genes in potato were retrieved by querying 269 nt sequence runs, covering the conserved BELL domain and the homeodomain of *StBEL5*, against the PGSC_DM_v3.4_gene.fasta file from the Potato Genome Sequencing Consortium website (http://potatogenomics.plantbiology.msu.edu). Based on genomic and expressed RNA sequence data, six new active *StBEL* genes, *StBEL6*, *-31*, *-32*, *-33*, *-34*, and *-35*, and one pseudogene were identified (Table 1). The open reading frames for these new BELs were predicted with FGENESH (http://linux1.softberry.com/berry.phtml) using the most closely related gene orthologue in tomato as a reference. Based on these amino acid sequences, a phylogenetic tree was constructed for the StBEL family (Fig. 1). BEL1-like proteins are characterized by four conserved regions: the SKY-box located in the N-terminal region, the BELL domain, the homeodomain, and the VSLTLGL motif in the C terminus (Supplementary Fig. S1 available at *JXB* online). The TALE (*t*hree-*a*mino acid *l*oop *e*xtension) is the proline-tyrosine-proline (PYP) link located between helices I and II.

**Table 1.** Sequence structure of 14 genes in the BEL1 family of potatoAn asterisk indicates the presence of a tandem TGAC-core motif in the promoter with no more than a 3 nt linker between the TGAC cores. CDS, coding sequence.

PGSC locus no.	gene	5′ UTR (nt)	Intron in 5′ UTR (nt)	CDS (aa)	3′ UTR (nt)
PGSC0003DMG400005930	BEL5*	149	203	2067 (688)	503
PGSC0003DMG400021323	BEL29	259	1893	2130 (710)	491
PGSC0003DMG400019635	BEL11	268	177	2130 (710)	317
PGSC0003DMG400010086	BEL13	388	470	2217 (738)	111
PGSC0003DMG400012329	BEL14	307	832	1902 (633)	76
PGSC0003DMG400022011	BEL22*	362	195	2088 (695)	74
PGSC0003DMG400030961	BEL30	466	966	1938 (645)	57
PGSC0003DMG400003751	BEL32	777	467	1986 (661)	414
PGSC0003DMG400024267	BEL33	234	None	1518 (505)	209
PGSC0003DMG400008057	BEL34*	54	None	2079 (692)	233
PGSC0003DMG400019142	BEL35	69	None	1728 (575)	109
PGSC0003DMG400029946	BEL6*	359	1090	1725 (574)	155
PGSC0003DMG400003750	BEL31	130	None	1272 (423)	175
PGSC0003DMS000003755	BEL15	pseudogene			

Phylogenetic analysis was structured on alignment of the StBEL1-like proteins with the 13 known members of the *Arabidopsis* BEL1 family (Rutjens *et al.*, 2009; Fig. 1). Overall the StBEL proteins clustered into five main clades that further branched into subclades. The six new BELs delineated into five independent branches of the phylogenetic tree with the closely related BEL33 and -35 clustered on the same branch (Fig. 1, arrows). In general, the BELs of potato matched very closely with their *Arabidopsis* orthologues.

**Fig. 2.**
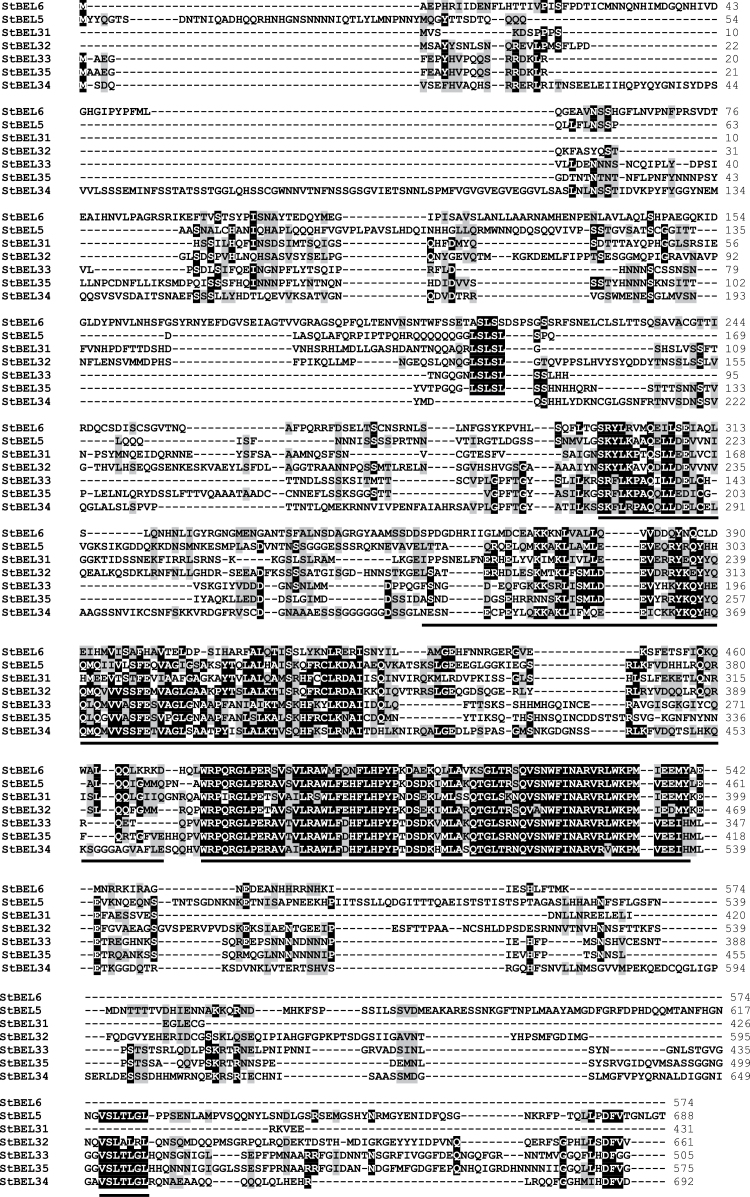
Amino acid sequence alignment of the six new StBELs with StBEL5. Black- and grey-boxed letters represent identical or similar residues, respectively. The conserved BELL domain (starting Leu272), homeodomain (starting Trp393) and the N-terminal SKY and C-terminal VSLTLGL boxes have been underlined. The amino acids for conserved domains are aligned in relation to the BEL5 protein.

**Fig. 3.**
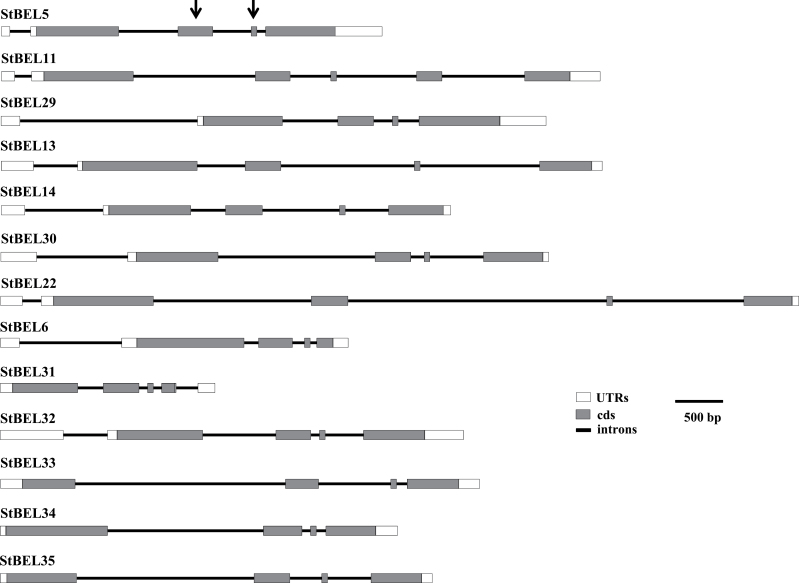
The structure of *StBEL* genes drawn to scale, according to the alignment of cDNA sequences against the corresponding genomic sequences. The cDNA sequence for the new StBELs was obtained by RT-PCR with gene-specific primers in combination with both 5′ and 3′ RACE and by utilizing genome sequence as needed. All genomic sequence for the *StBEL* family was obtained from sequence data from the Potato Genome Sequencing Consortium website (http://solanaceae.plantbiology.msu.edu). The conserved internal second and third exons representing coding sequence are indicated by arrows on the BEL5 gene structure.

**Fig. 4.**
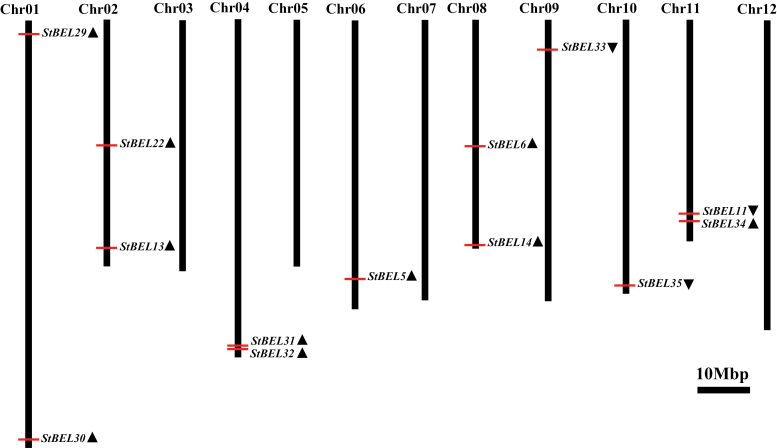
Genomic distribution of the *StBEL* genes on potato chromosomes. Chromosome numbers are shown at the top of each bar. The triangles following the gene names indicate the direction of transcription. The position (bp) of each *StBEL* gene on PGSC potato chromosome pseudomolecules (Release: Annotation v3.4, Assembly v3, Pseudomolecules v2.1.11) is specified in Supplementary Table S1 available at *JXB* online.

The amino acid sequences of the StBEL proteins range from 423 for BEL31 to 738 for BEL13 (Table 1) and displayed a range of divergence outside the four conserved regions (Fig. 2). Although conserved sequence motifs like LSLSL and DFV were evident towards the N and C termini, respectively, their functional significance is unknown (Fig. 2). BEL6 and -31 were relatively short BEL1-like proteins at 574 and 423 aa, respectively, and did not contain either the VSLTLGL or the DFV C-terminal motifs (Fig. 2). One other *StBEL* gene (*BEL15*, locus no. PGSC0003DMS000003755) was phylogenetically related to *StBEL14* and *-22*, but its ORF encoded a truncated protein structure and no expressed sequence tags were identified for it, suggesting it is inactive. The overall gene structure of this family was highly conserved. Twelve of the 13 active *StBEL* genes contained three or four introns. *StBEL11* contained five (Fig. 3). Twelve of the 13 also had four exons. Again, *BEL11* was the exception with five. Scoring UTRs, exons, and introns, 10 of the 13 genes ranged in length from approximately 4.0kb (*BEL5* and *-34*) to 6.3kb (*BEL11* and *-13*). *BEL31* and -6 were 2.3 and 3.65kb, respectively. *BEL22* was approximately 8.4kb. The length of the second and third coding sequence exons were conserved throughout the family (Fig. 3, arrows). Splicing appeared to be consistent at these four internal exonic junctions to produce sequences ranging from 353 to 411 nt for exon 2 and 61 nt for exon 3. For all potato BELs, exon 2 encoded an amino acid sequence that spanned a portion of both the BELL domain and the homeodomain. The third exon encoded a sequence in the homeodomain. The 3′ end of exon 2 contained 2 nt (CC) of the codon that encodes the first proline in the PYP TALE, whereas the 5′ end of exon 3 contained the other nucleotide (any of the four bases) of this proline codon. BEL34 was the lone exception with the complete proline codon present at the end of exon 2 and only 60 nt in exon 3. This exonic splicing pattern in the middle of the PYP TALE is conserved in tomato, rice, and *Arabidopsis* BEL1 genes. The 13 active BEL genes were distributed over eight of the 12 potato chromosomes (Fig. 4). No more than two genes were located on any one chromosome. The close proximity of *BEL31* and *-32*, only 2021bp apart on chromosome 4 (Supplementary Table S2 available at *JXB* online), suggested a recent tandem duplication event.

Because of their importance in regulating RNA mobility (Banerjee *et al.*, 2006, 2009), UTRs were scored for length by using the longest sequence obtained either by rapid amplification of cDNA ends (RACE) or from available web-based RNA sequence data. BEL5, -11, -29 and -32 had 3′ UTRs ranging from 317 to 503 nt, the four longest in the family (Table 1). Common sequence motifs were observed in the 3′ UTR sequences of BEL5, -11, and -29 (Supplementary Fig. S2 available at *JXB* online). BEL6, -13, -22, -30 and -32 contained the longest 5′ UTR sequences, ranging from 359 to 777 nt. Several polypyrimidine clusters of at least 3 nt were identified in the 3′ UTRs of BEL5, -11, and -29 (Supplementary Fig. S2 available at *JXB* online) and in the 5′ UTRs of BEL13, -14, and -30. These motifs are recognized by the polypyrimidine tract-binding proteins, an important class of RNA-binding proteins (Auweter and Allain, 2008; Ham *et al.*, 2009; Mahajan *et al.*, 2012). Intronic sequences ranging from 177 to 1893 nt interrupted the 5′ UTRs of all the BELs except BEL31, -33, -34, and -35 (Table 1).

### Expression patterns of potato BEL1-like genes

**Fig. 5.**
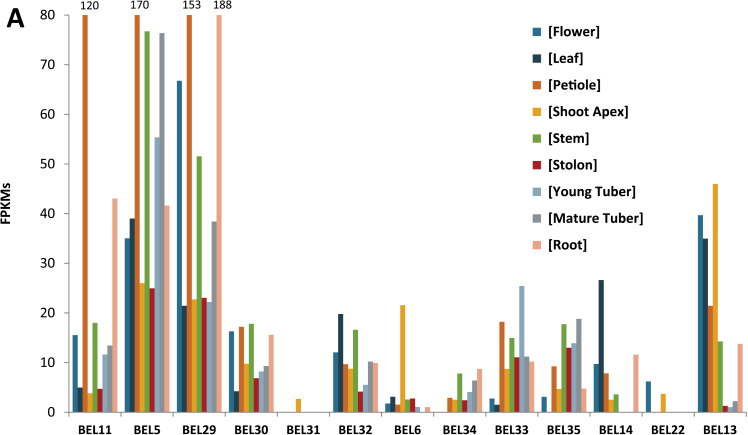

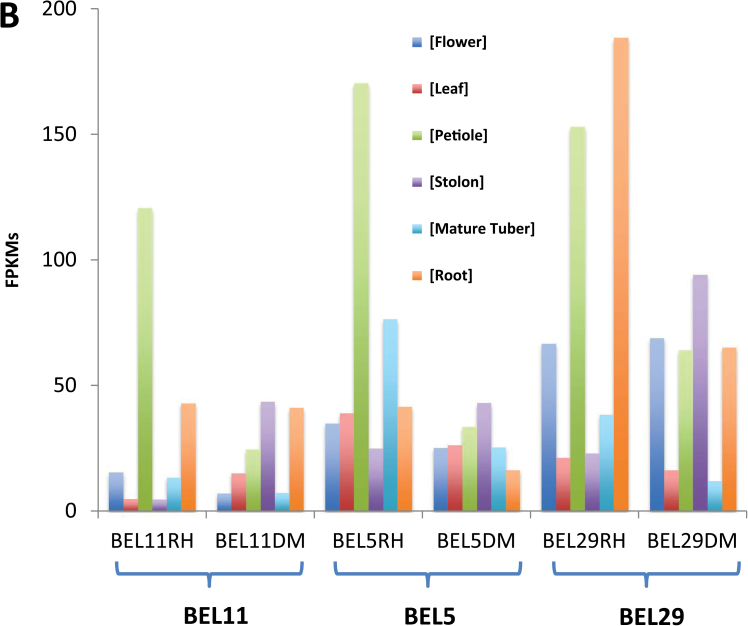
(A) Expression profile of StBEL family members mined using the RNA-seq data from the publically available Potato Genome Database from the Tuberosum RH89-039-16 haplotype (Xu *et al.*, 2011). Nine organs are presented and abundance values are shown in FPKMs (fragments per kb per million mapped reads). (B) A comparison of expression profiles of *StBEL11*, *-5*, and *-29* mined using the RNA-seq data from the publically available Potato Genome Database from both the RH and the DM1-3516-R44 haplotypes (Xu *et al.*, 2011). Six organs available from the DM database are presented for comparison and abundance values are shown in FPKMs.

Using RNA-Seq data of *S. tuberosum* group Tuberosum RH89-039-16 from the recently published potato genome (Xu *et al.*, 2011) and RT-PCR, widespread ubiquitous accumulation of most *StBEL* transcripts was generally observed (Fig. 5A and Supplementary Fig. S3 available at *JXB* online). With the RNA-Seq data, however, detectable fragment counts were observed in only two organs for *StBEL31* (sprouts and shoot apices) and *-22* (flowers and shoot apices). The most striking feature of this overall RNA expression profile, however, was that three of the *StBEL* genes (*StBEL5*, *StBEL11*, and *StBEL29*) exhibited a very high proportion, greater than two-thirds, of the overall RNA accumulation values for the entire StBEL family (Fig. 5A). *BEL5* was the most abundant RNA in six of the 10 organs and placed in the top three ranking for all organs (Supplementary Table S3 available at *JXB* online). *BEL29* was most abundant in three others. The one exception was in the shoot apex where *StBEL13* was most abundant. Overall, the 12 highest fragments per kb per million mapped reads (FPKMs) values compiled for all organs were for *BEL5*, *-11*, and *-29*,and these three accounted for 22 out of 30 of the top three abundance values in each organ category (Supplementary Table S3 available at *JXB* online). All three registered relatively high transcript values for petioles (170, 121, and 153, respectively) and stems (77, 17, and 52, respectively), both prominent organs involved in the transport of mobile signals (Banerjee *et al.*, 2006; Ham *et al.*, 2009). As an example, *StBEL5* mRNA is transcribed in leaf veins and petioles and moves into sieve elements of the phloem of both and is transported via the stem to stolon tips and roots to regulate growth (Banerjee *et al.*, 2006; Lin *et al.*, 2013). No transcription was observed for *StBEL5* in stems despite the high accumulation of its RNA detected in this organ (Banerjee *et al.*, 2006). The abundant and ubiquitous nature of *StBEL5*, *-11*, and *-29* RNAs, particularly in the petiole, and their phylogenetic similarity, suggest that they may act in a network of mobile RNA signals that regulates development throughout the plant. In roots, values were greatest for *StBEL5*, *-11*, and *-29* at 42, 43, and 188 FPKMs, respectively (Supplementary Table S3 available at *JXB* online). In tuber sprouts, their values topped out at 60, 46, and 62, respectively. The value of 188 for *BEL29* in roots was the greatest observed among all potato *BELs* in any organ. In the less robust (smaller tubers, smaller plants) *S. tuberosum* group Phureja DM1-3 516 R44 haplotype (Xu *et al.*, 2011), total transcript values for *BEL5*, *-11*, and *-29* were much less than in the RH haplotype, making up only 55% of total transcript values compared with 71% of the RH total abundance values (Fig. 5B). Abundance values of the other 10 *StBEL* RNAs were essentially the same in the two genotypes (Supplementary Fig. S4 available at *JXB* online).

**Fig. 6.**
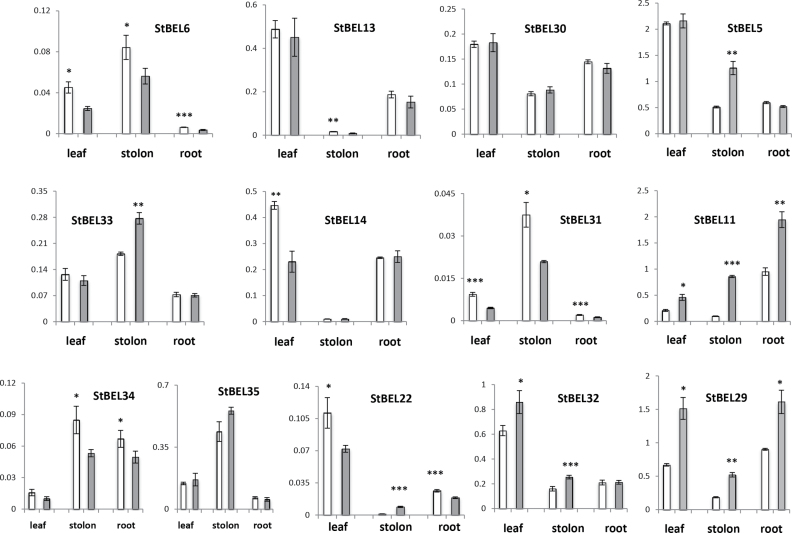
Effect of photoperiod on *StBEL* RNA accumulation in the photoperiod-responsive potato species, *S. tuberosum* ssp. *andigena* in leaves, stolon tips, and roots grown for 4 weeks. Relative levels of *StBEL* transcripts are presented on the *y*-axes and were quantified using total RNA extracted from new leaves (leaf), 0.5cm samples from the tip of stolons (stolon), or secondary roots (root) from plants grown under long (open bars) or short (grey shaded bars) days. SD plants were harvested after 10 d of SD conditions (8h light, 16h dark). Real-time qRT-PCR with gene-specific primers was used to calculate the relative amounts of RNA for each *StBEL* gene. The expression of each *BEL* gene was calculated as the 2^−ΔCt^ value and normalized to the endogenous reference gene, *StAct8.* The StBELs are organized phylogenetically by columns into four groups (see Fig. 1). Standard errors of the means of three biological replicates are shown with asterisks indicating significant differences (*P<0.05; **P<0.01; ***P<0.001, respectively) using Student’s *t*-test.

Previous work on the accumulation of *StBEL5* RNA showed that its abundance and mobility was regulated by photoperiod but that its transcription was induced by SDs only in stolons (Chen *et al.*, 2003; Banerjee *et al.*, 2006; Chatterjee *et al.*, 2007). Real-time qRT-PCR was performed on all members of the StBEL family to determine their RNA accumulation patterns in leaves, stolons, and roots in response to day length in the photoperiod-responsive *S. tuberosum* ssp. *andigena* (Fig. 6). *StBEL5*, *-11*, *-22*, -*29*, *-32*, and *-33* displayed upregulation in one or more organs from SD plants, whereas *StBEL6* and *-31* exhibited increased levels in all three organs under LDs. *StBEL34* RNA levels increased in both stolons and roots under LDs. No photoperiod effect was observed for either *StBEL30* or *-35*. *StBEL5*, *-11*, and *-29* exhibited the strongest induction in RNA accumulation in stolons in response to SDs. *StBEL6*, *-31*, *-33*, *-34*, and *-35* exhibited proportionately more RNA in stolons than leaves, whereas only a trace of RNA for *StBEL13*, *-14*, and *-22* was detected in stolons (Fig. 6). The relative abundance levels of these RNAs were generally consistent with the RNA-Seq data (Fig. 5A) with *StBEL6*, *-22*, *-31*, and *-34* being the least abundant and *StBEL5*, *-11*, and *-29* being the most abundant among the *StBEL* genes. Clearly, a diverse range of transcript concentrations was evident in these StBEL family members. For example, the relative abundance difference between transcript levels in SD leaves for *StBEL5* and *-31* was approximately 480-fold.

### Interaction with KNOX partners

The BEL1-like homeodomain proteins interact physically with their KNOX homeodomain protein partners to regulate gene expression by controlling the transcription of target genes (Bellaoui *et al.*, 2001; Müller *et al.*, 2001; Smith *et al.*, 2002; Chen *et al.*, 2003, 2004). The seven originally identified members of the StBEL family displayed selective interaction with the Knotted1-like protein, POTH1 (Chen *et al.*, 2003), and four other tobacco class I-type KNOX (NTH1, -15, -20 and -22) proteins (Supplementary Table S4 available at *JXB* online). To test for the interaction of the new BEL1-like gene family members with Knotted1-type proteins, all of the six new BELs and three other previously identified StBELs (StBEL5, -13, and -30) representative of a wide phylogenetic range across the StBEL family, were evaluated for protein interaction in the yeast two-hybrid system.

**Fig. 7.**
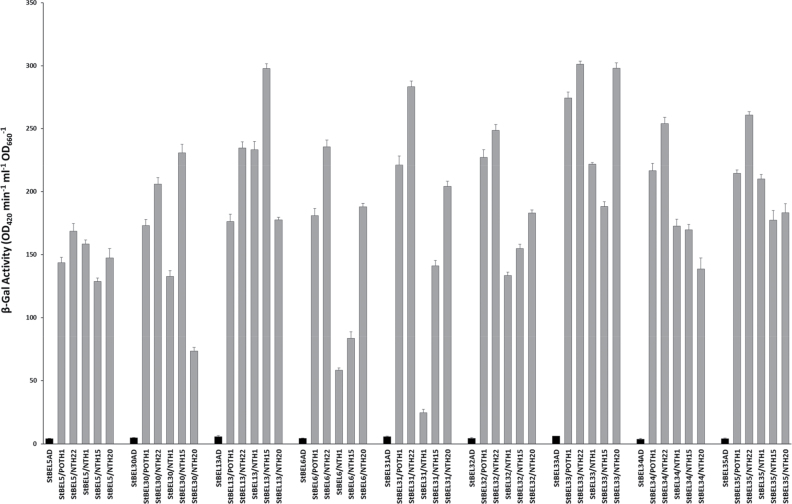
Specific interaction of POTH1 and four KNOTTED1-type proteins of tobacco with nine BEL1-like proteins, BEL5, -30, -13, -6, -31, -32, -33, -34 and -35, of potato using a β-galactosidase assay to assess the strength of interaction. The new potato BELs were cloned into the pACT-AD vector and the KNOTTED1-types were expressed in pBridge. BEL5/POTH1-BD was used as a reference and the new BEL proteins in the pACT-AD vector transformed into yeast are shown as negative controls. *LacZ* induction in the yeast strain pJ69-2A was assayed in transformed yeast cultures using a quantitative yeast β*-*galactosidase assay method. Standard errors of the means of three replicate samples are shown for each combination.

Interaction was tested with POTH1 and the four tobacco KNOX types and quantified using β-galactosidase activity (Fig. 7). NTH22 is the tobacco orthologue of POTH1. All of the potato BEL1-like proteins displayed an interaction with all five KNOX proteins but their binding affinities varied considerably. The NTH22 interaction with StBEL33, for example, based on β-galactosidase activity was the strongest among all the StBEL proteins tested (301 Miller units), whereas the interactions between NTH1 and StBEL31 and -6 were the weakest (25 and 58 Miller units, respectively). Overall, StBEL interactions with NTH1 exhibited some of the lowest levels of β-galactosidase activity with an average of 149 Miller units per interaction. Among the KNOX types, NTH22 exhibited the greatest activity levels with an average of 243 units per interaction. Interactions with StBEL13, -33, and -35 had the strongest overall interactions with the five KNOX types. Among the StBEL proteins, the most robust interactions with NTH1 and -15 (SHOOTMERISTEMLESS orthologue) were with StBEL13 (233 and 298 units, respectively), whereas the strongest interactions with POTH1, NTH22, and NTH20 were with StBEL33 (274, 301, and 297 units, respectively). StBEL5, -6, -30, and -31 displayed the weakest interactions overall with these KNOX partners.

### Tandem TTGAC motifs in the StBEL gene family

**Fig. 8.**
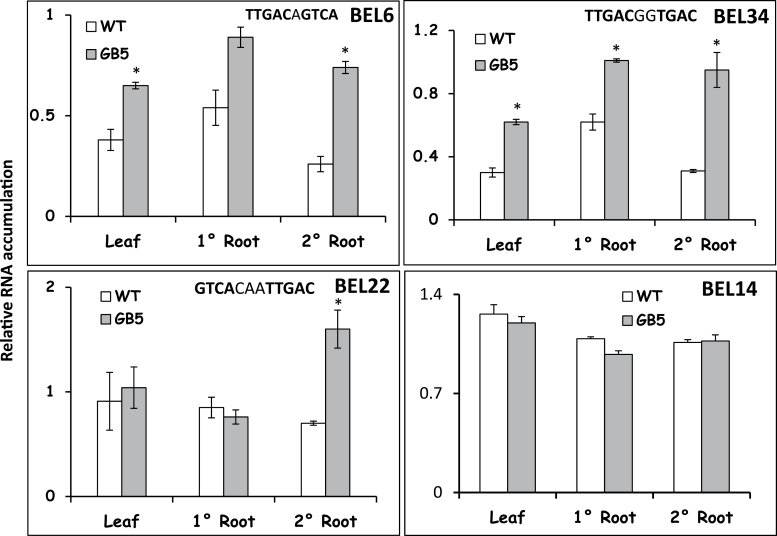
Cross-regulation of endogenous *StBEL6*, *-34*, and *-22* in *GAS:BEL5* overexpression lines. The movement of transgenic *StBEL5* mRNA from leaf to primary and secondary roots was confirmed previously using transgenic lines expressing full-length *StBEL5* RNA driven by the GAS promoter of melon (*Cucumis melo*) grown under SD conditions (Fig. 1B; Lin *et al.*, 2013). This promoter is expressed in the minor veins of leaf mesophyll (Ayre *et al.*, 2003; Banerjee *et al.*, 2009). Substantial amounts of transgenic *BEL5* RNA moved into primary and secondary roots and activated accumulation of WT *StBEL5* transcripts (Fig. 7; Lin *et al.*, 2013). This same RNA was used to assess levels of endogenous RNA for *StBEL6*, *-34*, *-22*, and *-14* in both the WT (open bars) and transgenic BEL5 line (grey shaded bars) in leaves, primary (1° Root) and secondary (2° Root) roots. The existing upstream double TGAC core motifs are shown for each gene. The *BEL14* upstream sequence contains no tandem TGAC motif and was included as a negative control. One-step RT-PCR was performed using 200–250ng of total RNA and gene-specific primers for *StBEL6*, *-34*, *-22*, and *-14*. All PCRs were standardized and optimized to yield product in the linear range. Homogenous PCR products were quantified using ImageJ software (Abramoff *et al.*, 2004) and normalized using 18S rRNA values. Standard errors of the means of three replicate samples are shown. The asterisk indicates a significant difference (*P*<0.05) using Student’s *t*-test.

StBEL5 functions in tandem with its KNOX partner, POTH1, to bind specifically to a 10bp sequence consisting of twin TTGAC core motifs to regulate developmental processes in potato (Chen *et al.*, 2003, 2004). Examination of the *StBEL5* promoter revealed inverted tandem TTGAC motifs spaced 3 nt apart in a head-to-head orientation on opposite stands 820 nt upstream from the start of its 5′ UTR. Using a mutated promoter driving GUS expression, this double motif was confirmed to be involved in mediating autoregulation of *StBEL5* in stolons and roots (Lin *et al.*, 2013). To check for the possibility of cross-regulation among the *StBELs*, upstream sequences up to 3kb for each *StBEL* gene were screened for TGAC motifs. Four of the 13 *StBEL* members, including *StBEL5*, harboured the TGAC core motif in tandem separated, at most, by a 3 nt linker (Fig. 8). *StBEL6* contained the motif in a tail-to-tail orientation on opposite stands (**TTGAC**a**GTCA,** 520 nt upstream from the start of the 5′ UTR). *StBEL22* had the motif in a head-to-head orientation again on opposite stands (**GTCA**caa**TTGAC,** 1471 nt upstream from the start of the 5′ UTR), whereas the motifs are present in a tail-to-head direction on the same (+) strand in *StBEL34* promoter sequence (**TTGAC**gg**TGAC,** 1459 nt upstream from the start of the 5′ UTR).

**Fig. 9.**
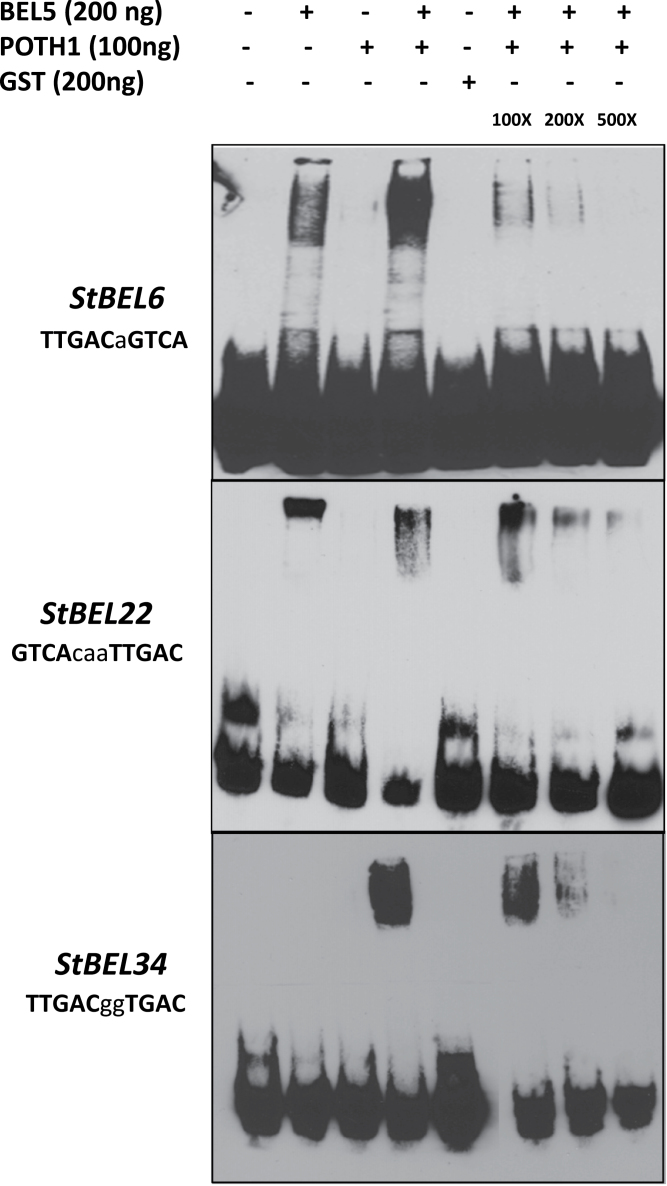
Gel-shift assays of various tandem TGAC core motifs (bold, upper-case nucleotides) in three putative target genes of StBEL5 and POTH1 with a range of linker sequence (lower-case nucleotides) between motifs. Upstream sequences of *StBEL6* contain the tandem motifs in a tail-to-tail orientation on opposite DNA strands and *StBEL22* motifs exhibit a head-to-head orientation, whereas *StBEL34* contains the tandem motifs in a tail-to-head orientation on the same (+) DNA strand. The StBEL5 and POTH1 proteins were expressed and purified with a C-terminal GST fusion tag. Each DNA bait was tested for binding with StBEL5–GST, POTH1–GST, or GST alone or with StBEL5–GST and POTH1–GST together. Ten femtomoles of synthesized DNA probes of 30 (*StBEL6*) or 50 nt labelled with biotin were used in the binding reaction. The amounts of StBEL5 and POTH1 proteins used in these assays were adjusted to achieve equivalent molarity. Unlabelled DNA bait at 100×, 200×, and 500× concentrations relative to the labelled probe was used in the competition assays.

Using gel-shift assays, tandem head-to-head and tail-to-head TGAC motifs present on the *StBEL5* and *StGA20ox1* promoters, respectively, were confirmed as binding targets to the StBEL5/POTH1 heterodimer (Chen *et al.*, 2004; Lin *et al.*, 2013). Double palindromic tail-to-tail TGAC core motifs present in upstream sequence of *StGA2 oxidase1* also bound to this tandem protein complex (Lin *et al.*, 2013). In all three of these examples, specificity of binding to the tandem TGAC element was confirmed through mutagenesis. To further study the significance of these motifs on the promoter activity of *StBEL6*, *-22*, and *-34*, RNA accumulation for these genes was assayed in a transgenic line that couples leaf-specific overexpression of *StBEL5* with the capacity to transport *BEL5* transcripts into stolons and roots (Banerjee *et al.*, 2006). Using the leaf-specific galactinol synthase (GAS) promoter (Ayre *et al.*, 2003), movement of *StBEL5* RNA from leaves to stolons and roots was readily observed with the greatest level of accumulation occurring in secondary roots (Lin *et al.*, 2013). In theory, any RNA driven by the GAS promoter (in this case, *StBEL5*) that is detected in organs other than the leaf is the result of long-distance transport. In this way, this system monitors the induction of a target gene by a mobile RNA signal. The relative expression patterns of *StBEL6*, *-22*, and *-34* were assayed in leaves, primary and secondary roots of the GAS:BEL5 transgenic line relative to non-transformed controls. RNA levels were enhanced 2- to 3-fold for all three *BEL* genes in secondary roots in correlation with transgenic *StBEL5* RNA accumulation (Fig. 8). Induction was also observed in leaves and primary roots for *StBEL34* and in leaves for *StBEL6*. No induction was observed in the *GAS:BEL5* transgenic plants in the levels of mRNA for *StBEL14*, a *StBEL* gene without a tandem TGAC motif present in its upstream sequences. DNA-binding assays confirmed the interaction of the StBEL5/POTH1 protein heterodimer with the double TGAC core motifs present in all three of these *StBEL* genes (Fig. 9). In the case of the *StBEL6* and *-22* motifs, a strong interaction with the StBEL5 protein alone was also observed. The tightly resolved band in the upper portion of lane two (BEL5 protein alone) for *StBEL22* suggested the presence of a homodimer. Three of the four 5′→3′ DNA strand orientations for the two core motifs are represented in this *StBEL* group: tail-to-tail, head-to-head, and tail-to-head on the (+)-strand (Table 2 and Fig. 9). In previous work, mutated forms of the identical tandem motifs present in *StBEL6*, *-22* and *-34* exhibited diminished binding to the StBEL5/POTH1 complex (Chen *et al.*, 2004; Lin *et al.*, 2013). As reported previously (Fig. 6), these three putative targets of the StBEL5 complex are among the rarest of the *StBEL* transcripts.

**Table 2.** Eight target genes of StBEL5The TGAC core motifs running 5′→3′ are in bold letters. Linker sequence between the motifs is shown in lower-case letters. The location of the motif is designated upstream from either the transcription (TSS) or the translation (AUG) start site.

Gene	Motif ^*a*^	Orientation^*b*^	Location of motif (nt upstream)	RNA levels regulated by StBEL5	Binding confirmed by EMSA^*c*^	Reference
*StBEL5*	**GTCAA**tgc**TTGAC**	HtH	820 (TSS)	Yes	Yes	Lin *et al.*, 2013
*StBEL6*	**TTGAC**a**GTCA**	TtT	520 (TSS)	Yes	Yes	Figs 8 and 9
*StBEL22*	**GTCA**caa**TTGAC**	HtH	1471 (TSS)	Yes	Yes	Figs 8 and 9
*StBEL34*	**TTGAC**gg**TGAC**	TtH ^(+)^	1459 (TSS)	Yes	Yes	Figs 8 and 9
*StGA20ox1*	**TTGACTTGAC**	TtH ^(+)^	700 (TSS)	Yes	Yes	Chen *et al.*, 2004
*StGA2ox1*	**TTGAC**aa**GTCA**	TtT	1768 (AUG)	Yes	Yes	Lin *et al.*, 2013
*StIPT*	**TTGAC**aa**GTCA**	TtT	1408 (AUG)	Yes	Yes	Hannapel *et al.*, 2013
*YUCCA1a*	**TTGAC**ctta**TTGAC**	TtH ^(+)^	641 (AUG)	Yes	Yes	Lin *et al.*, 2013

^*a*^ The criteria for these motifs was the inclusion of at least one TTGAC and one TGAC on either strand of the DNA with a linker sequence of no more than four nt.

^*b*^ Three 5′→3′ orientations were observed: head-to-head (HtH), tail-to-tail (TtT), or tail-to-head on the plus strand (TtH^(+)^). No double motifs aligned tail-to-head on the (–) strand were identified in this initial screen.

^*c*^ Verified binding to the BEL5/POTH1 complex via EMSA. Stronger binding with the BEL5/POTH1 complex was observed than with either protein alone.

## Discussion

### Targets of the StBEL transcription factors

To date, members of the BEL1-like family of transcription factors have been identified in every plant species that has so far been studied. With the advent of full-genome sequences, the breadth and potential functions of this key family of DNA-binding proteins may now be fully understood. Clear evidence has established the role of the BELs in both floral and vegetative development. A catalogue of the known target genes for BEL1 transcription factors supports this premise. Most prominent in this currently minute collection are *GA20 oxidase1*, *GA2 oxidase1*, *YUCCA1a*, *isopentenyl transferase*, *StBEL5*, *PIN1 and -2* (Chen *et al.*, 2004; Lin *et al.*, 2013; Hannapel *et al.*, 2013), and of course, as shown here, other *BEL1*-like genes (Table 2). All of these targets contribute to important aspects of plant growth, including meristem maintenance, tuberization, and leaf and root development. BEL1 proteins may also play important roles in response to biotic stress and pathogen challenge (Luo *et al.*, 2005) and in regulating lignin biosynthesis (Mele *et al.*, 2003). The strong wound response exhibited by the promoter of *StBEL5* (Chatterjee *et al.*, 2007) suggests that BEL1 proteins may function in defence against abiotic stress.

### The *StBEL5/11/29* clade

These three StBEL types group phylogenetically in a unique cluster with *AtBLH1* of *Arabidopsis*. AtBLH1 functions with KNAT3 to affect establishment of cell fates in the mature embryo sac (Pagnussat *et al.*, 2007). Each of these three StBEL proteins was among the largest proteins in the potato group (688 aa for BEL5 and 710 for both BEL11 and -29) and contained conserved amino acid sequence domains outside the canonical motifs. Their overall transcript abundance levels were consistent and unique. All three exhibited relatively high levels in petioles, stolons, roots, and tuber sprouts, whereas *StBEL5* and *-29* exhibited high levels in flowers, shoot apices, and young and mature tubers (Supplementary Table S3 available at *JXB* online). All three exhibited enhanced levels of RNA accumulation in stolons from SD plants (Fig. 6). Together, these observations suggested that *StBEL5*, *-11*, and *-29* are relatively stable RNAs that play pivotal roles in regulating development in actively growing organs. Within this group, *StBEL5* functions as a mobile RNA that impacts growth in both tubers and roots (Banerjee *et al.*, 2006; Lin *et al.*, 2013). As discussed previously, the RNA metabolism of *StBEL5* is mediated by its 3′ UTR (Banerjee *et al.*, 2006, 2009), and there are sequences within this region that are common to both *StBEL11* and *-29* (Supplementary Fig. S2 available at *JXB* online). It is conceivable that StBEL members of this subgroup are functionally redundant and share a similar long-distance, non-cell-autonomous delivery system.

### Levels of regulation controlling *StBEL* gene activity

Perhaps the most intriguing aspect of the StBEL family is its complex mode of regulating expression and activity at both the transcriptional and post-transcriptional levels. StBEL5 regulates activity of its own promoter (Lin *et al.*, 2013), and in the current study, movement and accumulation of transgenic *BEL5* RNA were also correlated with an increase in steady-state levels of three other *StBEL* RNAs. This increase was observed only with genes containing the tandem core TGAC motif recognized by the BEL/KNOX complex (Smith *et al.*, 2002; Chen *et al.*, 2004; Lin *et al.*, 2013). Auto- and cross-regulation among plant transcription factors in the same family is now known to be quite common. MADS genes are regulated by MADS-box proteins in a wide network of protein–DNA interaction. SEPALLATA3 binds to *cis*-regulatory elements of other MADS-box genes and is a key component in the transcriptional network regulating the formation of floral organs (Kaufmann *et al.*, 2009). Positive autoregulation of Knox genes in rice was essential for shoot apical meristem development (Tsuda *et al.*, 2011). In this study, OSH1 directly bound to five KNOX loci (including itself) to upregulate expression. Using ChIP-seq in maize, Bolduc *et al.* (2012) showed that KN1 directly targets upstream sequence of numerous transcription factors, including its own gene, nine other KNOX types, and five BEL1-like genes. Two of the target maize BEL1-like genes are orthologues of *StBEL6* and *-34*. As a mechanism for enhancing specificity, it is very likely that many of these maize DNA interactions are mediated by BEL/KNOX tandem complexes. Gel-shift assays with native DNA sequences of potato showed that binding of BEL/KNOX complexes was consistently stronger than with either protein alone (Fig. 9; Chen *et al.*, 2004; Lin *et al.*, 2013; Hannapel *et al.*, 2013).

At the post-transcriptional level, BEL1-like proteins exhibit several potential mechanisms for the control of activity or expression. These include, first, the availability and binding affinity of protein partners. These partners may include KNOX proteins, ovate family proteins (OFPs), or a MADS-box homeodomain protein complex that contains the SEPALLATA MADS-box proteins (Brambilla *et al.*, 2007). Interaction with KNOX proteins facilitates selective transport of the tandem complex into the nucleus (Bhatt *et al.*, 2004). In a similar manner, the ovate family proteins, AtOFP1 and AtOFP5, associate with the cytoskeleton and interact with both BEL and KNOX proteins to regulate their subcellular localization to the cytoplasm (Hackbusch *et al.*, 2005). By preventing nuclear localization, the OFPs essentially block BEL/KNOX activity. In another example of partner interaction, a truncated form of a KNOX protein of *Arabidopsis*, designated KNATM-B, encodes a MEINOX domain but not the homeodomain (Magnani and Hake, 2008). This new class of KNOX proteins is conserved in eudicots, including both tomato (Magnani and Hake, 2008) and potato (PGSC0003DMP400031538) and selectively interacts with BEL proteins through the MEINOX domain. These results suggest that KNATM-B may prevent specific BEL proteins from taking part in transcriptional complexes by sequestering them in an inactive dimer or by localization in the cytoplasm.

Secondly, the binding affinity of the BELL/KNOX complex for the various tandem TGAC motifs can regulate activity. As shown previously, binding may occur to double elements with tail-to-tail, head-to-head, or (+)- and (–)-strand tail-to-head orientations (Table 2; Chen *et al.*, 2004; Hannapel *et al.*, 2013; Lin *et al.*, 2013). Very little is known about how these various configurations affect the interaction of the BEL/KNOX or KNOX/KNOX complexes with the upstream target *cis*-element. Such differences in binding affinity could certainly impact the results on cross-regulation of *StBEL6*, *-22*, and *-34* presented here (Figs 8 and 9) as each of these promoters contain a unique configuration of the tandem core TGAC element.

The third post-transcriptional mechanism that can significantly affect BEL1 activity is the non-cell-autonomous nature of BEL1-like mRNAs. Specific *StBEL* RNAs are transcribed in one organ and have the capacity to move long distances via the phloem to target organs. The best example of a mobile RNA in the BEL1 family is *StBEL5*. RNA movement assays demonstrated that *StBEL5* transcripts move through the phloem to stolon tips to regulate tuber formation. *StBEL5* mRNA originates in the leaf and its movement to stolons is induced by a SD photoperiod (Banerjee *et al.*, 2006). Movement of *StBEL5* RNA into roots correlated with increased growth and the accumulation of several transcripts associated with hormone metabolism has also been reported (Hannapel *et al.*, 2013; Lin *et al.*, 2013). Regulated long-distance transport of full-length mRNAs is a unique signalling process and represents a dynamic mechanism to separate transcription and translation, and in this case, to control both the temporal and spatial activity of a pivotal transcription factor. Based on RNA profiling in phloem cells (Yu *et al.*, 2007; Campbell *et al.*, 2008), it is very likely that other *StBEL* genes (like *StBEL11* and *-29*) may function in a similar manner.

## Supplementary data

Supplementary data are available at *JXB* online.

Supplementary Fig. S1. Schematic protein structure of StBELs.

Supplementary Fig. S2. Alignment of the 3′ UTRs of *StBEL11* and *-29* to *StBEL5*.

Supplementary Fig. S3. RT-PCR for select *StBEL* genes.

Supplementary Fig. S4. Expression profile for StBEL family members using RNA-Seq data.

Supplementary Table S1. List of primers and oligonucleotides.

Supplementary Table S2. Position of *StBEL* genes on PGSC chromosome pseudomolecules.

Supplementary Table S3. RNA-Seq data for 13 *StBEL* genes.

Supplementary Table S4. Interaction of tobacco KNOX and potato BEL1 proteins.

Supplementary Data

## Abbreviations:

FPKM: fragments per kb per million mapped reads
GAS: galactinol synthase
GUS: β-glucuronidase
GST: glutathione *S*-transferase
LD: long day
NTH: *Nicotiana tabacum* homeobox
qRT-PCR: quantitative reverse transcription-PCR
RACE: rapid amplification of cDNA ends
SD: short day
UTR: untranslated region
WT: wild type.
