# Supplementary material for: The BEL1-like family of transcription factors in potato
Source: J Exp Bot. 2014 Jan 27;65(2):709–23. doi: 10.1093/jxb/ert432 (PMC3904721; doi:10.1093/jxb/ert432)
Supplement: Supplementary Data [file supp_65_2_709__index.html]

The BEL1-like family of transcription factors in potato — Supplementary Data 

# The BEL1-like family of transcription factors in potato

## Supplementary Data

Data files

**Files in this Data Supplement:**

- Supplementary Data - Supplementary Data
