# Supplementary material for: The BEL1-like family of transcription factors in potato
Source: J Exp Bot. 2014 Jan 27;65(2):709–23. doi: 10.1093/jxb/ert432 (PMC3904721; doi:10.1093/jxb/ert432)
Supplement: Supplementary Data [file supp_ert432_jexbot108571_file001.pdf]

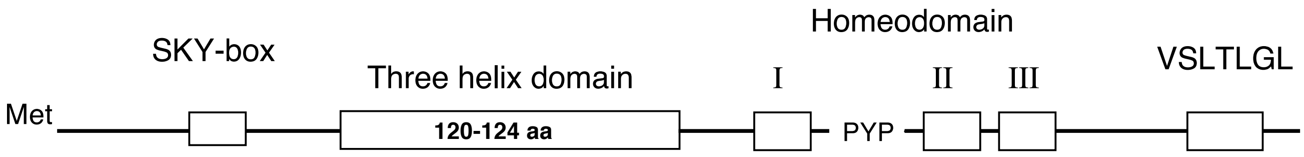

Figure S1. Protein structure of StBELs. All StBELs contain a SKY box, a BELL domain and a homeodomain made up of three helices. A conserved three aa loop extension (TALE), proline-tyrosine-proline (PYP), is located between the first and second helices. The VSLTLGL motif is conserved but its function is unknown.

```
BEL11     ------------UAUAUAUAAUUUGCAGGUAAAUCAGCUUGAAA------------UUAC
BEL5      AUACCAGAAAGUCUCGUAUUGAUAGCUGAAAAGAUAAAAGGAAGUUAGGGAUACUCUUAU
                       : .***:.:*:**:*.:**.: *..: ***.            ***

BEL11     AUCAUGAAAGGCCUU-------------------------------------GAAUAAAA
BEL5      AUUGUGUGAGGCCUUCUGGCCCAAGUCGGAGGACCCAAUUUGAUACAACCUAUCAUAGGA
          ** .**:.*******                                      .***..*

BEL11     GAAGGGGAGUUGAGAUCUAGUGAUCAUAUAAAUAUGUAUAGGUAGAAAGUUUAGUUAGUA
BEL5      GAAAAGAAGUGGAGACUAAAUUAAAGUAACAAAAUUUUAAAGCACACUUUCUAGUAUAUA
          ***..*.*** ****  :*.* *:..**:.**:** *::*.* * *.: * ****::.**

BEL11     UA----------------------------------------------------------
BEL5      UACUUCUUUUUUUUAUAGUAUAGAAAAGAAGAGAUUUUGUGCUUUAGUGUAUAGAUAGAG
          **

BEL11     ---------UAUAGGUUAUACUUCUAGUUUCUUAAAUGGA--GAUACAAUUUUUGUUGUU
BEL5      UCUACUUAGUAUAGGUUAUACUUCUAGUUCCUUGAGAAGAUUGAUACAACUAGUAGUAUU
                   ******************** ***.*.:.**  ******* *: *. *.**

BEL11     AUUUUUGUAUUGAGAUAACUAG-----CUAGCUUGGAUUAUU------------------
BEL5      UUUUUUCUUUUGGGUUGGCUUGGAGUACUAUUUUAAGUUAUUGGAAACUAGCUAUAGUAA
          :***** *:***.*:*..**:*     ***  **...*****

BEL11     --------------UAAAGUUGUUGCAUGCAACCAAAGAAGAAGAAAAAAUAAUCUAUAU
BEL5      AUGUUGUAAAGUUGUGAUAUUGUUCCUCUCAAUUUGCAUAUAAUUUGAAAUAUUUUGUAC
                        *.*:.***** *:  ***  :...:* ** ::.*****:* *.**

BEL11     AUGCAAACUAUAGUAUGUUGUAAAUUUUGUGCGUCU----------UUUUGUUUCAAUUU
BEL5      CUACUAGCUAGUCUCUAAAUUAUGUUUCCAUUGCUUGUAAUUGCAAUUUUAUUUGAAUUU
          .*.*:*.*** : *.*.:: **:.***  :  *  *          ****.*** *****

BEL11     ---------------------------GCAUAUAUGUAAAC
BEL5      UGUGCUAUCAUUAUUAGAUUAGCAAAAAAAAAAAAAAAAAA
                                     ..*:*:*:..:***.
```

```
BEL29     UCACAAAAACAAAAACAGGUUUUGGCAACAGACAAACUUCUGUCGCUAAACAAGGACAUG
BEL5      AUACCAG---AAAGUCUCGUAUUGAUAGCUGA-------------------AAAGAUAAA
          : **.*.   ***.:*: **:***. *.*:**                   **.** *:.

BEL29     AUUUAGCGACAGAUAACUUCAGUCGCUAACUUAGCGACUGAAAACUUCUGUCGCUAAGCA
BEL5      AGGAAGUUAGGGAUACUCUUA----------UAUUGUGUGAGGCCUUCUGGCCCAAGUCG
          *  :**  * .****.  * *          **  *: ***...****** * *:*. *.

BEL29     UGAACAUGUAUUAGCGACA------UACAGUAUGCAACUGUAUGUCACUAAACAAGAACA
BEL5      GAGGACCCAAUUUGAUACAACCUAUCAUAGGAGAAAAGAAGUGGAGACUAAAUUAAA--G
           .....  :***:*. ***       * **  * ..** :.  : *: ****** :*.*  .

BEL29     UGAUGAAUUAGUGACGGACAACUUCUGUCGC---------------------UAAACAAC
BEL5      UAACAAAAUUUUAAAGCACACUUUCUAGUAUAUAUACUUCUUUUUUUUUAUAGUAUAGAAA
          *.* .**:*: *.*.* ***. ****.   .                     **:* **.

BEL29     AAAAAAAAAUCCAUGUUUUAGUAUAUUGUU--UCUCAUUCUAUCAUAUCAUGGUAGUGUA
BEL5      AGAAGAGAUUUUGUGCUUUAGUGUAUAGAUAGAGUCUACUUAGUAUAGGUUAUACUUCUA
          *.**.*.*:*  .** ******.***:*:*  : **::  **  ***  :*. :. * **

BEL29     AAGAAUCAAGAAACAAGUUUUACAUAGUAACAGUCUUUAUACAUUG----GAGAUGAAGA
BEL5      GUUCCUUGAGAAGAUUGAUACAACUAGUAGUAUUUUUUUUCUUUUGGGUUGGCUUGGAGU
          .: ..* .****..::*:*: *..*****. * * ***:*. :***    *. :**.**:

BEL29     ACCA-UUUAAGUUCUUCAAA-------AUAGAUAGAUUUUCUAGGUUAC-------UUCU
BEL5      ACUAUUUUAAGUUAUUGGAAACUAGCUAUAGUAAAUGUUGUAAAGUUGUGAUAUUGUUCC
          ** * ********.** .**       ****::*.: **  :*.***.        ***

BEL29     ACAAGAUAUAUAUAUGGUUGAGGGU-UUGUAUAUUAAUUUUGGGAUUGUUAUAUUGGAUG
BEL5      UCUCAAUUUGCAUAUAAUUUGAAAUAUUUUGUACCUACUAGCUAGUCUCUAAAUUAUGUU
          :*:..**:*. ****..** ....* ** *.**  :* *:   ..*   **:***. .*

BEL29     UGGAAAAAAAGUAGUUAUUUUGGGUGGUAUAAAUAAAAUAAUACUCCAUCCAUUUUAGCC
BEL5      UCCAUUGCUUGUAAUUGCAAU--UUUAUUUGAAUUUUGUGCUAUCAUUAUUAGAUUAGCA
          *  *::..::***.**. ::*   * .*:*.***:::.*..**  . ::  * :*****.
```

Figure S2. Alignment of the 3' UTRs of StBEL11, and -29 to StBEL5. Conserved sequences to StBEL5 are shown in gray highlight and bold letters. Polypyrimidine motifs in BEL11 and -29 sequence are shown bold and underlined. There are nine such CU motifs (at least, 3 nt in length) in BEL11 and fourteen in BEL29.

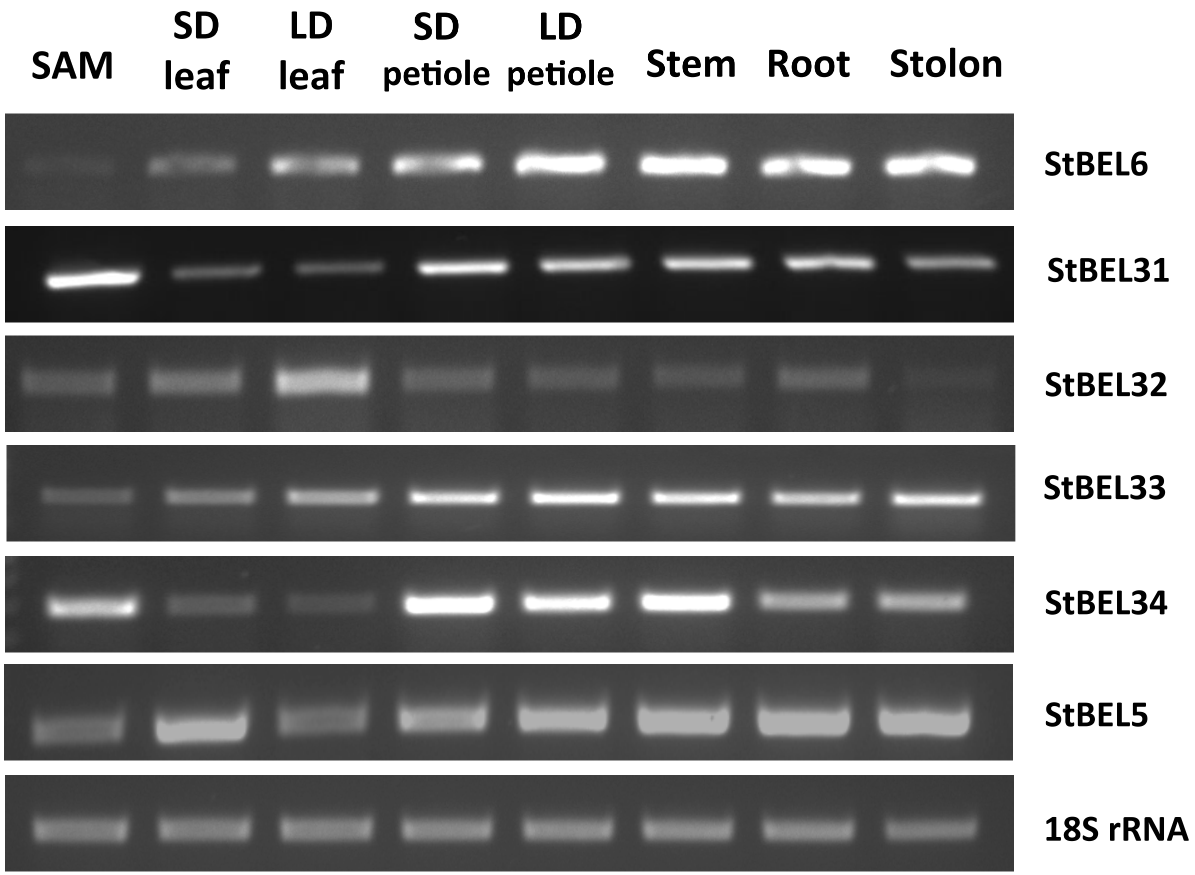

Fig. S3. RT-PCR with gene-specific primers for *StBEL5*, *-6*, *-31*, *-32*, *-33* and *-34* using total RNA extracted from the shoot apical meristem (SAM), leaf (SD and LD), petiole (SD and LD), stem, root and stolon of soil-grown potato plants, *Solanum tuberosum* ssp. *andigena*. RT-PCR product for 18S rRNA was used as an internal control.

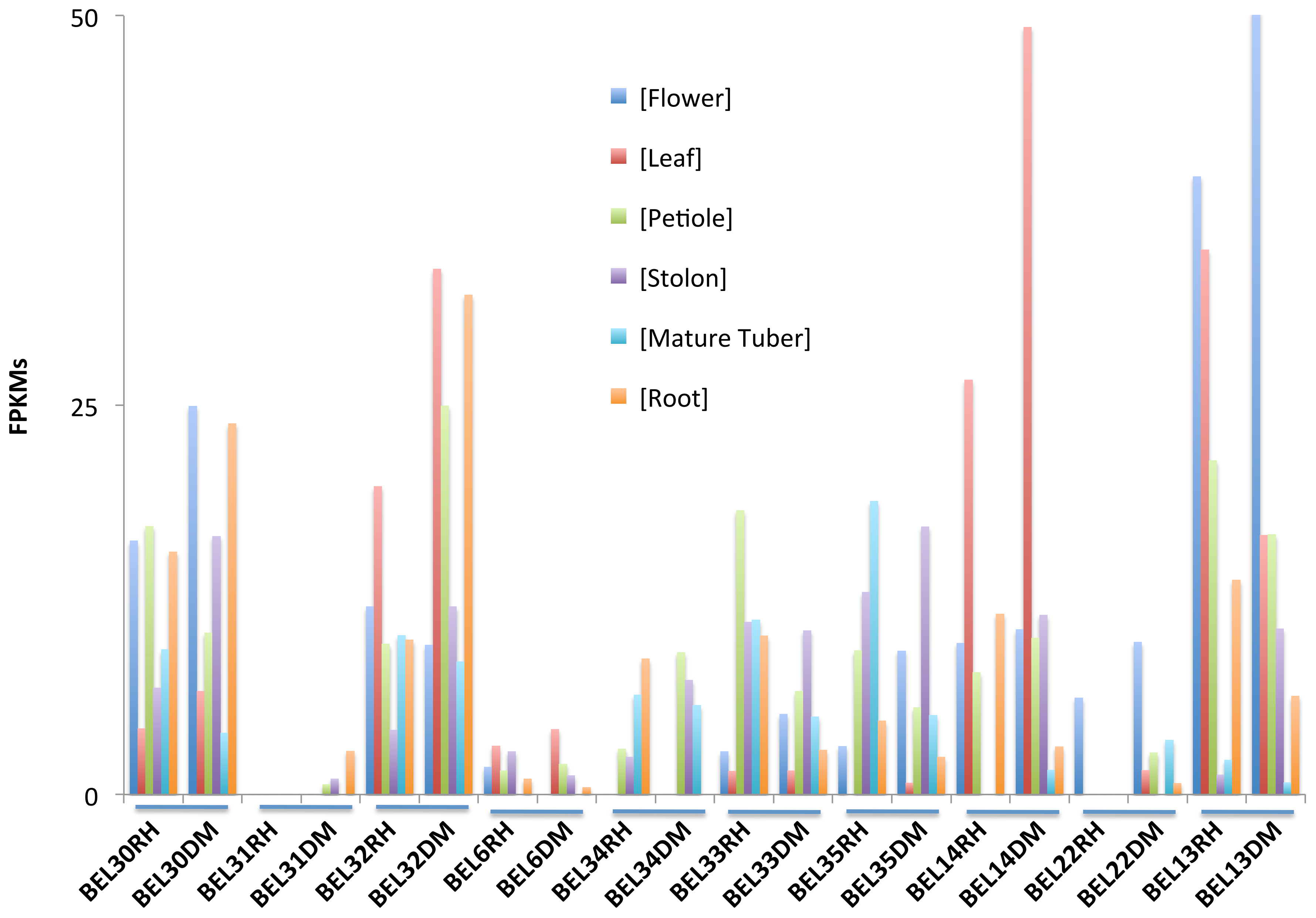

Figure S4. Expression profile for StBEL family members mined using the RNA-seq data from the publically available Potato Genome Database from both the RH and DM genotypes (Xu *et al.*, 2011). Six organs and ten StBELs are presented and abundance values are shown in FPKMs (fragments per kb per million mapped reads).

Table S1. Primers and oligonucleotides

For qRT-PCR of BELs LD/SD

BEL5-F: AGGGATACTCTTATATTGTGTGAGG
BEL5-R: GTCTCCACTTCTTTTCTCCTATG
BEL6-F: GGATGCAGAGAAGCAATTGCTGG
BEL6-R: TGGTTTCCATAGACGAACTCGAG
BEL31-F: CTCCATCCATATCCAAATGACTC
BEL31-R: CGTTAATAAACCAGTTGGAAACCTGA
BEL32-F: TGCTGTTTCAGTCCTTCGTGCTT
BEL32-R: AACCTGTTTGCCTCGCCAGCATGAT
BEL33-F: CTTCCTGAACGTGCTGTCACTGTA
BEL33-R: CATTACTTTATCGCTGTCCGTGG
BEL34-F: GCACATGTTGGCTTCTCAAAC
BEL34-R: CATCCGATTTTCTTGTTTGATCTC
BEL35-F: GACTGGTCTTTCAAGGAATCAG
BEL35-R: GTGTATTTCTTCAACCATAGGC
Actin-F: GGAAAAGCTTGCCTATGTGG
Actin-R: CTGCTCCTGGCAGTTTCAA

For cloning Y2H constructs

StBEL6fw: CATGCCATGGAG**ATG**GCAGAGCCTCATCGG
StBEL6rev: TCCCCCCGGGTCACTTCATTGTAAATAAATG

StBEL31fw: GAATTCGAATGGTTTCAAAAGACTCACCCC
StBEL31rev: CTCGAGTCATTCTTCTGCAGAATCAGTAACAC

StBEL32fw: CATGCCATGGAG**ATG**TCTGCTTACTATTCAAATTTG
StBEL32rev: TCCCCCCGGGTTACACTACAAAATCGGATAG

StBEL33fw: CATGCCATGGAG**ATG**GCTGAAGGATTTGAAC
StBEL33rev: TCCCCCCGGGTCAACCACCAAAATCATGC

StBEL34fw: CGGGATCCGA**ATG**GTGGCTTTTTGCGATG
StBEL34rev: CCGCTCGAGTCAATCAACAAAGTCATG

StBEL35fw: GAATTCGAATGGCTGCAGAAGGATTTGAA
StBEL35rev: CTCGAGTCAACCAACAAAATCATGCAAC

GSPs for BEL6, -14, -22, and -34 of Fig. 8

BEL6Fw: GTATGCTGAGATGAATAGAAG
BEL6Rv: GGCATAGCATACAACATGG

BEL34Fw: CGGATGTGAATAAGCTCGTG
BEL34Rv: CAGCTCCGATATTACCTCC

18SFw: GGATGTTGCTTTTAGGACTC
81SRv: CATCACAGACCTGTTATTGC

BEL22Fw: ATTGTCGCAATGAATACACATTA
BEL22Rv: TTATGTAGCAGTGTTTGAATACAC

BEL14Fw: TATGGAAGCCAATGGTGGAAG
BEL14Rv: ATGATCCACCACTTCCACCAT

Oligos for EMSA DNA bait 6, 22, and 34 for Fig. 9

BEL6S: TGCTTGCCTTTTGACAGTCACTATTGTATG
BEL6A: CATACAATAGTGACTGTCAAAAGGCAAGCA

BEL22S: TATGTCCGTTGCATAATTAGTCACAATTGACATGCTCCTATTAGATATAA
BEL22A: TTATATCTAATAGGAGCATGTCAATTGTGACTAATTATGCAACGGACATA

BEL34S: CCTTTCAATAAGATTATTGACGGTGACAATACTCGCCGTCAAA
BEL34A: TTTGACGGCGAGTATTGTCACCGTCAATAATCTTATTGAAAGG

Table S2

Chr no.	Size of chr	BEL gene location		
chr01	81.48 Mbp	(BEL29) 3.678 kbp from chr01, positions 2,458,978 to 2,462,655	(BEL30)5.85 kbp from chr01, positions 79,264,769 to 79,270,618	
chr02	47.07 Mbp	(BEL13)7.139 kbp from chr02, positions 43,219,985 to 43,227,123	(BEL22)5.199 kbp from chr02, positions 23,380,997 to 23,386,195	
chr03	47.88 Mbp	none		
chr04	64.34 Mbp	(BEL31)2.258 kbp from chr04, positions 62,325,726 to 62,327,983	(BEL32)4.917 kbp from chr04, positions 62,330,004 to 62,334,920	bel31 and 32 are 2.021 kbp apart, tandemly duplicated genes?
chr05	47.05 Mbp	none		
chr06	54.98 Mbp	(BEL5)4.015 kbp from chr06, positions 49,229,263 to 49,233,277		
chr07	53.43 Mbp	none		
chr08	43.65 Mbp	(BEL14)4.946 kbp from chr08, positions 43,062,646 to 43,067,591	(BEL6)2.659 kbp from chr08, positions 24,072,529 to 24,075,187	
chr09	53.64 Mbp	(BEL33)5.086 kbp from chr09, positions 5,515,256 to 5,520,341		
chr10	52.31 Mbp	(BEL35)4.593 kbp from chr10, positions 50,859,636 to 50,864,228		

chr11	42.25 Mbp	(BEL34)4.122 kbp from chr11, positions 38,532,179 to 38,536,300	(BEL11)6.483 kbp from chr11, positions 37,602,233 to 37,608,715	**BEL34 and 11 are 0.923 Mbp apart**
chr12	59.1 Mbp	none		

BEL	Gene	Flower	Tuber Sprout	Leaf	Petiole	Shoot Apex	Stem	Stolon	Young Tuber	Mature Tuber	Root
31	PGSC0003DMG400003750	0	2.5	0	0	2.7	0	0	0	0	0
32	PGSC0003DMG400003751	12	10.3	19.8	9.7	8.8	16.6	4.2	5.5	10.2	9.9
**5**	**PGSC0003DMG400005930**	**35**	**60**	**39**	**170.3**	**25**	**76.7**	**24**	**55.4**	**76.4**	**41.6**
34	PGSC0003DMG400008057	0	15.1	0	2.9	2.5	7.8	2.4	4.1	6.4	8.7
13	PGSC0003DMG400010086	**39.7**	4.1	**34**	21.4	**45**	14.2	1.3	1	2.2	13.8
14	PGSC0003DMG400012329	9.7	2.7	**26.6**	7.8	2.51	3.6	0	0	0	11.6
35	PGSC0003DMG400019142	3.1	20.8	0	9.2	4.7	17.7	**12**	13.9	**18.8**	4.7
**11**	**PGSC0003DMG400019635**	15.5	**46.4**	4.9	**120.6**	3.8	17	4.7	11.6	13.4	**43**
**29**	**PGSC0003DMG400021323**	**66.8**	**61.5**	21.4	**153**	**22.7**	**51.5**	**23**	**22.2**	**38.4**	**188.4**
22	PGSC0003DMG400022011	6.2	0	0	0	3.7	0	0	0	0	0
33	PGSC0003DMG400024267	2.8	20.6	1.5	18.2	8.7	14	11.1	**25.4**	11.2	10.2
6	PGSC0003DMG400029946	1.8	1.9	3.1	1.5	21.6	2.6	2.8	1.1	0	1
30	PGSC0003DMG400030961	16.3	14.9	4.2	17.2	9.8	**17.8**	6.8	8.2	9.3	15.6

Table S3. RNA-seq data for thirteen *StBEL* genes from the RH potato genotype (Xu *et al*., 2011). BEL1-like protein coding genes were identified, and the FPKM value of each is shown for several organs. Bold numbers represent the top three values in each category. *StBEL5* is the most abundant RNA in 6 of 10 organs. *BEL29* is most abundant in three others. *BEL5* and *BEL29* are top two most RNAs abundant in nine of the organs.

Table S4. Interaction of select tobacco KNOX proteins with potato BELs verified with *β-galactosidase* assays in the yeast two-hybrid system. The plus sign indicates strong *β-gal* induction; the negative sign, no induction. The NTH Knox genes 1, 15, 20, and 22 are from *Nicotiana tabacum*. POTH1 and the class II-Knox are from potato.

Knox type	BEL5	BEL11	BEL13	BEL14	BEL22	BEL29	BEL30
class II-Knox	–	–	–	–	–	–	+
POTH1	+	+	+	+	+	+	+
NTH1	+	+	+	+	+	+	+
NTH15	+	+	+	+	+	+	+
NTH20	+	+	+	+	+	+	+
NTH22	+	+	+	+	+	+	+
